# Current Status of Extended Adjuvant Endocrine Therapy in Early Stage Breast Cancer

**DOI:** 10.1007/s11864-018-0541-1

**Published:** 2018-04-27

**Authors:** Irene E. G. van Hellemond, Sandra M. E. Geurts, Vivianne C. G. Tjan-Heijnen

**Affiliations:** Division Medical Oncology, GROW – School of Oncology and Developmental Biology, Maastricht University Medical Centre, P.O. Box 5800, 6202 AZ Maastricht, The Netherlands

**Keywords:** Endocrine therapy, Aromatase inhibitor, Tamoxifen, Adjuvant therapy, Breast cancer, Postmenopausal

## Abstract

In the past decade, several endocrine treatment regimens have been developed for the adjuvant treatment of postmenopausal women with hormone receptor-positive early breast cancer, including tamoxifen, aromatase inhibitors (AI), or a combination of these. The standard duration of adjuvant endocrine treatment has been 5 years for a long time. Nevertheless, the high number of recurrences occurring after 5 years suggested that extended endocrine therapy could further improve outcome, which led to the start of several randomized clinical trials investigating the effects of extended use of endocrine therapy. The extended duration of tamoxifen has been shown to improve disease-free survival and overall survival in the ATLAS and aTTom trials. However, in postmenopausal women, AIs have been shown to be more effective when compared with tamoxifen. Based hereon, it is recommended that adjuvant endocrine therapy in postmenopausal women with early breast cancer should include an AI. Recently, the DATA, IDEAL, and NSABP B42 trials showed that extended adjuvant endocrine therapy with AIs beyond 5 years in postmenopausal women with early breast cancer did reduce the occurrence of secondary breast tumors, but had no or only a small impact on distant metastasis free survival. Furthermore, toxicity of adjuvant AIs led to gradually decreasing compliance rates and long-term toxicities to non-breast cancer-related deaths. Therefore, we suggest considering extended adjuvant treatment only in women with high-risk early breast cancer who tolerate treatment well.

## Introduction

For years, tamoxifen has been the standard adjuvant endocrine treatment of hormone receptor-positive breast cancer in both pre- and postmenopausal women. In the past decade, several other treatment regimens have been developed, using tamoxifen, aromatase inhibitors (AIs), or a combination of these.

The latest ASCO guideline regarding adjuvant endocrine therapy provides clear recommendations on extended tamoxifen treatment for premenopausal women with hormone receptor-positive early breast cancer [1]. However, for postmenopausal patients, a choice remains between four different treatment regimens; AI monotherapy for 5 years, sequenced treatment with tamoxifen and AIs for 5 years, extended tamoxifen monotherapy for 10 years, or tamoxifen followed by extended AIs for 10 years. To determine which 5-year schedule showed the highest efficacy, the Early Breast Cancer Trialist’s Cooperative Group (EBCTCG) has compared three treatment strategies in the adjuvant setting of early breast cancer in postmenopausal women: continuous AI versus tamoxifen monotherapy, sequential tamoxifen and AI versus tamoxifen monotherapy, and sequential tamoxifen and AI versus continuous AI monotherapy [2••]. Their meta-analysis showed that 5-year adjuvant endocrine treatment including AIs was more effective than tamoxifen monotherapy in preventing recurrence and breast cancer death in either continuous or sequential regimens.

Recently, a number of trials have been published where the efficacy and tolerability of extended endocrine therapy with AIs beyond 5 years were studied [3•, 4•, 5•, 6•]. In this present review, we aim to summarize published randomized controlled trials on the efficacy and tolerability of different regimens of adjuvant endocrine therapy in postmenopausal women with hormone receptor-positive early breast cancer. In particular, the available evidence in terms of efficacy and tolerability of extended adjuvant endocrine treatment beyond 5 years. Moreover, we discuss potential difficulties and consequences of extending endocrine treatment in daily practice in subgroups of postmenopausal women with early breast cancer.

## Methods

A detailed search strategy was used to search the PubMed database, consisting of numerous MeSH heading and text word combinations, “breast cancer,” “endocrine therapy,” “tamoxifen,” “aromatase inhibitors,” “exemestane,” “anastrozole,” “letrozole,” “adjuvant,” “extended,” and “postmenopausal.” Publications of randomized clinical phase III trials published before December 2017 in English language were included in our analysis. Abstracts of the yearly conferences of the San Antonio Breast Cancer Symposium (SABCS) and the American Society of Clinical Oncology (ASCO) were searched for relevant trials (and substituted by full papers if published before December 2017). Furthermore, we scanned the references of relevant trials, existing meta-analyses and guidelines for additional important trials. We categorized the studies by treatment regimen (tamoxifen, AI, or sequential) and duration (up to 5 years or more than 5 years). Studies concerning locally advanced and/or metastatic disease were excluded.

Hazard ratios (HR) were used to assess the treatment effects in each trial. If available, the HRs were directly obtained from the published article or conference presentation. If the trials did not provide HRs, they were calculated using the available methods of Tierney and colleagues [7]. When the results of the included trials were published at multiple points in time, the results with the longest follow-up duration were included.

## Efficacy of treatment up to 5 years

The first randomized trials of adjuvant endocrine treatment for early breast cancer started in the mid-1970s and compared 1 to 2 years of tamoxifen with no endocrine treatment showing a reduction in breast cancer recurrences in the tamoxifen treatment groups [8–10]. The observation that these recurrences seemed to occur mostly after the adjuvant treatment period, with a median follow-up of 44–66 months, led to the hypothesis that a longer duration of treatment would further improve outcome.

In the early 1980s, a multicenter randomized trial demonstrated the superiority of 5 years of adjuvant tamoxifen over 2 years in the treatment of postmenopausal women with hormone receptor-positive early breast cancer [11]. This additional benefit in terms of breast cancer recurrence and mortality was confirmed by later trials and meta-analyses [12–14].

Later on, AIs were developed, offering an alternative strategy to tamoxifen in the adjuvant treatment of postmenopausal women with hormone receptor-positive early breast cancer by preventing the production of endogenous oestrogens. The ATAC trial and the BIG 1-98 trial were the first large trials comparing adjuvant AIs with tamoxifen each for a duration of 5 years in postmenopausal women [15, 16]. Anastrozole was used in the ATAC trial and letrozole in the BIG 1-98 trial. AIs were found to be superior to tamoxifen in terms of disease-free survival (DFS), recurrence-free survival (RFS), and overall survival (OS).

Thereafter, several studies were performed to investigate the effect of sequenced treatment, using different approaches, but all comparing with 5 years of tamoxifen [17–21]. The ABCSG-8 trial randomized patients between sequential tamoxifen followed by AIs or continuous tamoxifen therapy immediately after the primary breast cancer treatment (surgery, chemotherapy, and/or radiation therapy) and showed a statistically significant improvement in DFS for patients treated with AIs (HR 0.78 (95% CI 0.60–1.00)). Moreover, OS improved, although not statistically significant (HR 0.87 (95% CI 0.64–1.16)) [22]. Other trials randomized patients after the initial treatment with tamoxifen, thus selecting a subpopulation of patients with possibly better prognosis and higher endocrine sensitivity [18–21]. Study findings should therefore be interpreted with caution. With the exception of the Japanese NSAS BC03 trial, all trials showed a statistically significant improvement in terms of DFS for sequential endocrine therapy in comparison with 5 years of tamoxifen. After a median follow-up varying between 30 and 128 months, all trials showed an improved OS, but these results were only statistically significant in the IES and ITA trials.

In addition, both the BIG 1-98 and the TEAM trial addressed the switch to an AI after 2–3 years of tamoxifen in comparison with AI monotherapy for a total of 5 years [16, 17, 23•]. These trials randomized patients directly after primary breast cancer treatment (surgery, radiation therapy, and/or chemotherapy). Neither study showed a preference for either strategy after a median follow-up of 8.1 and 9.8 years, respectively. This was in line with the intention-to-treat patient-level meta-analysis by the EBCTCG showing that both continuous and sequential regimens including AIs are more effective than tamoxifen monotherapy for 5 years in preventing recurrence and breast cancer death [2••]. AI monotherapy was associated with a significant 30% reduction in recurrences during the first year of endocrine treatment, when compared with a sequential regimen with tamoxifen followed by an AI. In the years thereafter, the number of recurrences did not differ between the treatment groups. Since it is expected that this benefit during the first year of endocrine therapy will not disappear, it is likely that, with longer follow-up, this benefit will also show in DFS and OS outcomes.

## Efficacy of extended treatment duration

Hormone receptor-positive breast cancer is characterized by a very long natural history. As a consequence, some women remain at risk of late recurrence for years, fueling the discussion to prolong endocrine therapy beyond 5 years. The risk of breast cancer recurrence after 5 years of endocrine therapy was evaluated in a meta-analysis by the EBCTCG [24••]. In that meta-analysis, breast cancer recurrences occurred at a steady rate throughout the study period from 5 to 20 years, strongly correlated with the original tumor- and nodal status and tumor grade. Among the patients with stage T1 disease, the risk of distant recurrence in the period from 5 to 20 years was 13% without nodal involvement (T1N0), 20% with N1-3 status, and 34% with N4-9 status; among those with stage T2 disease, the risks were 19% with T2N0, 26% with T2N1-3, and 41% with T2N4-9. The risk of death from breast cancer was similarly dependent on TN status.

Other studies reported an annual rate of distant relapse in excess of 2% for at least 15 years after diagnosis, even after 5 years of tamoxifen [25]. A similar risk remains for at least 10 years for postmenopausal women who have received AIs for 5 years [15]. The Oxford overview analyses likewise show that at least 50% of recurrences occurred more than 5 years after diagnosis [13]. To determine whether there is any outcome advantage in continuing adjuvant endocrine therapy for more than 5 years, and what the optimal duration of adjuvant endocrine treatment is, several strategies have been researched. These trial findings are summarized in Table 1 and are discussed next.

**Table 1:**
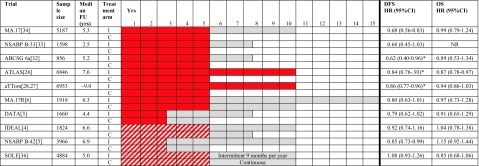
Overview of the reported results considering efficacy in the published trials on extended adjuvant endocrine treatment in postmenopausal women with early stage breast cancer

Red: tamoxifen. Gray: aromatase inhibitor. Diagonal lines: either tamoxifen or an aromatase inhibitor.

*FU* follow-up, *I* intervention arm, *C* control arm, *yrs.* years, *DFS* disease-free survival, *HR* hazard ratio, *CI* confidence interval, *OS* overall survival

*No data on DFS available, data on RFS reported

### Extended tamoxifen monotherapy

Results from the recent large ATLAS and aTTom trials clearly demonstrated that 10 years of tamoxifen showed an improved RFS and OS in comparison with 5 years of tamoxifen treatment (no data about DFS available) [26–28]. Also, the smaller ECOG trial showed a benefit for 10 years tamoxifen treatment [29]. In contrast, data from the NSABP B-14 trial and the Scottish trial failed to demonstrate a positive impact of prolonged tamoxifen treatment on RFS and OS [30, 31].

### Extended sequential regimen

Additionally, there have been studies investigating the use of AIs after 5 years of tamoxifen treatment. The ABCSG-6a, MA.17 and NSABP B33 trials all showed a clear benefit of 5 years AI treatment after an initial 5 years of tamoxifen in DFS [32–34]. There was also an improvement in OS, but this was not statistically significant. This may have been caused by a lack of power due to early unblinding of the study. The early interim analysis of the MA.17 trial, after a median of 2.5 years of follow-up, showed an improved DFS for women using letrozole after 5 years of tamoxifen (HR 0.58 (95% CI 0.45–0.76)). After unblinding, 60% of placebo patients crossed over to letrozole, which complicated the efficacy analysis. Due to the results of the MA.17 interim analysis, also the NSABP B33 trial was unblinded early after a median follow-up of 2.5 years.

Several studies investigated the efficacy and safety of additional treatment with AIs after a sequential regimen of tamoxifen and an AI for 5 years [3•, 4•, 5•]. The DATA trial investigated the effectiveness of 6 versus 3 years of anastrozole after an initial 2–3 year of tamoxifen in patients who were disease-free 3 years after randomization [3•]. The 5-year adapted DFS was not statistical significantly better for the overall study population in the 6-year group (HR 0.79 (95% CI 0.62–1.02); *p* = 0.066). However, in the subgroup of women with high-risk tumors, extended endocrine therapy was associated with an improved adapted DFS. For instance, in women with node positive disease, 5-year DFS was 84% in the 6-year group versus 76% in the 3-year group (HR 0.64 (95% CI 0.46–0.89), *p* = 0.0075); and 83% versus 69% if also having a larger tumor size (≥ T2; HR 0.53 (95% CI 0.53–0.82), *p* = 0.0031). Nevertheless, great care must be taken when interpreting subgroup analyses and should be interpreted as hypothesis generating rather than definitive. The results of a similar trial (GIM-4-LEAD; NCT01064635) are awaited, studying the effect of letrozole for 2–3 years versus 5 years after an initial 2–3 years of tamoxifen.

The IDEAL trial investigated the use of 2.5 versus 5 years of letrozole after an initial 5 years of endocrine treatment [4•]. The initial treatment could either be tamoxifen monotherapy, AI monotherapy, or a sequential regimen. Regardless of the initial treatment regimen, no statistically significant benefit on DFS and OS was found for 5 years of extended letrozole treatment in comparison to an extended 2.5 years of AI treatment.

The NSABP B42 study investigated the efficacy of 5 years of letrozole after an initial 5-year of endocrine therapy including an AI [5•]. This could be either AI monotherapy, or sequenced with tamoxifen. In the overall analysis, no statistically significant benefit was found for extended letrozole on DFS and OS. However, the results for distant recurrence-free survival (DRFS) and breast cancer-free interval (BCFI) were statistically significantly better for the extended treatment group (DRFS: HR 0.72 (95% CI 0.53–0.97), *p* = 0.03; BCFI: HR 0.71 (95% CI 0.56–0.89), *p* = 0.003).

The MA.17R trial investigated the efficacy of 5 years of letrozole after an initial 10-year treatment with tamoxifen for 5 years followed by AI for 5 years [6•]. The 5-year DFS rate was 95% with letrozole and 91% with placebo (HR 0.80 (95% CI 0.63–1.01), *p* = 0.06). The rate of 5-year OS was not different (93 versus 94% for the letrozole and placebo groups respectively). The annual incidence rate of contralateral breast cancer in the letrozole group was 0.21%, and the rate in the placebo group was 0.49% (HR 0.42 (95% CI 0.22–0.81), *p* = 0.007). This suggests that the benefit of extended endocrine therapy in this trial was mainly caused by a reduction in the development of contralateral breast cancer.

In a recent meta-analysis on extended endocrine therapy, including the abovementioned trials, particularly women with a positive nodal status seemed to have more benefit of extended endocrine therapy (node positive HR 0.72 versus node negative HR 0.83) [35•]. Similarly, a relative larger benefit was seen from extended endocrine therapy in women with a larger tumor size (> 2 cm HR 0.77 versus ≤ 2 cm HR 0.88), and for those with both ER and PR expression versus single receptor expression (HR 0.68 versus 1.01). A greater effect was also seen in patients who received adjuvant chemotherapy compared with those who did not (HR 0.71 versus 0.80). However, as exposure to chemotherapy is probably a surrogate measure for worse disease, this finding could be a reflection of higher chemotherapy receipt among patients with larger tumors and/or nodal involvement. Even though the differences in effect size of AIs between the higher and lower risk groups were not statistically different, it is yet an intriguing observation as in contrast to these AI studies, extended tamoxifen yielded similar relative benefits for the prognostic subgroups [26].

Another treatment approach was tested in the SOLE trial, in which it was hypothesized that resistance to letrozole could be reversed by withdrawal and reintroduction of letrozole [36•]. Postmenopausal women, previously treated by 5 years of endocrine treatment (tamoxifen, AI, or sequential), were randomized to either 5 years of intermittent letrozole or 5 years of continuous letrozole. Intermittent letrozole use did not improve DFS compared with continuous letrozole use (HR 1.08 (95% CI 0.93–1.26)).

## Compliance

Compliance is an important issue in adjuvant endocrine therapy in general because it influences the efficacy. A recent analysis of the BIG 1-98 trial looked at treatment adherence and its impact on DFS in patients on tamoxifen, letrozole, or a sequential regimen for 5 years [37]. Both early cessation and a low compliance score were associated with a reduced DFS. Sequential treatments were associated with higher rates of non-persistence (Tam-Let, 20.8%; Let-Tam, 20.3%; Tam 16.9%; Let 17.6%). In 82.7% of patients, adverse events were the reason for discontinuation. The reason sequential endocrine therapy with tamoxifen and AIs could be preferred over AI monotherapy is diverse. Costs, due to patency, used to play a restricting role in the use of AIs. Nowadays, adverse events like musculoskeletal events and bone loss are frequently the motivation for switching therapies [38, 39]. Furthermore, Henry and colleagues reported a 32% discontinuation rate for initial AI therapy within 2 years due to adverse events; 24% of the total study population discontinued specifically because of musculoskeletal symptoms [40]. The high percentage of discontinuation in the women taking tamoxifen might be explained by a younger age. A large cohort study published by Hershman and colleagues reported that women aged under 40 years had the highest risk of discontinuation in comparison with older aged women (HR 1.51 (95% CI 1.23–1.85) [41]. Also, two other studies showed a younger age to be a predictor of premature discontinuation of tamoxifen [42, 43].

For both tamoxifen and AIs, the probability of early termination increases with a longer treatment duration. A systematic, qualitative meta-regression analysis illustrated endocrine treatment discontinuation rates ranging from 31 to 73% over the treatment period [44]. In the women taking tamoxifen, 13.6% discontinued during the first year of treatment, which increased to 47.1% at 5 years. In the women taking AIs, percentages of discontinuation were 11.7% during the first year and 31.3% at 5 years [44, 45]. Likewise, another study described increasing discontinuation rates each year of AI treatment, ranging from 14 to 22% in the first year to 21–38% in the third year [42]. Early discontinuation rates in the published trials investigating extended endocrine therapy are as high as 30% [3•, 4•, 5•, 37].

## Tolerability

Each type of endocrine therapy is known for its drug-specific side effects. Tamoxifen inhibits the growth of breast tumors by competitive antagonism of estrogen at its receptor site. Its actions are complex and it also has partial estrogen agonist effects. These partial agonist effects can be beneficial, since they may help prevent bone demineralization in postmenopausal women, but also unfavorable, as they are associated with increased risks of uterine cancer and thromboembolism [38].

AIs suppress plasma and intra-tumoral estrogen concentrations in postmenopausal women by inhibiting or inactivating aromatase: the enzyme responsible for synthesizing oestrogens from androgenic substrate [46]. Unlike tamoxifen, AIs have no partial agonist activity. AIs have side effects that are predominantly predictable consequences of estrogen deprivation [38]. Musculoskeletal events (e.g. arthralgia and myalgia), bone loss and cardiovascular events have been reported frequently during AI use [39]. In contrast to tamoxifen, follow-up of the adjuvant AI trials is relatively short- and the long-term consequences of adjuvant AI use have yet to be fully determined.

A meta-analysis including seven trials comprising 16,349 patients analyzed the reported toxicity of extended endocrine treatment with AIs [47•]. Longer treatment with AIs was associated with increased odds of cardiovascular events (odds ratio (OR) = 1.18, *p* = 0.05, number needed to harm (NNH) = 122), bone fractures (OR = 1.34, *p* < .001, NNH = 72), and cessation of treatment due to adverse events (OR = 1.45, *p* < 0.001, NNH = 21). Extended use of AIs did not influence the odds of a second malignancy (OR = 0.93, *p* = 0.56), but a numerical excess of deaths without breast cancer recurrence was found with prolonged AI (OR = 1.11, *p* = 0.34). Even though the increase of deaths without breast cancer recurrence was not statistically significant, this might change when future results of these trials with a longer follow-up duration are published. The updated results of the TEAM trial, comparing 5 years of anastrozole with a 5-year sequenced regimen with tamoxifen and anastrozole with a median follow-up of 9.8 years, showed that the potential beneficial effect of exemestane on breast cancer-specific mortality might be counterbalanced by an increase in non-breast cancer-related mortality (12 versus 10%), leading to a similar overall survival between the treatment groups [23•].

## Postmenopausal due to prior chemotherapy

AIs are contraindicated in premenopausal women. Noteworthy, AIs are also contraindicated in women with chemotherapy-induced ovarian function failure because of the possibility of ovarian function recovery [48, 49]. Therefore, we advise against using AIs in women with chemotherapy-induced ovarian function failure, and also advise caution even when used in combination with gonadotropin-releasing hormone (GnRH) agonists. GnRH agonists do not suppress the ovarian function completely in all patients, as was observed in the SOFT-EST trial [50]. During 12 months of follow-up, 34.2% of the patients had inadequately suppressed E2 levels, at least once, indicating incomplete ovarian function suppression [50]. This might be the underlying reason that the combination of AI/GnRH agonist has not shown to improve overall survival in comparison with tamoxifen monotherapy or the combination of tamoxifen/GnRH [51, 52]. Hence, for women who became postmenopausal due to prior chemotherapy, extended adjuvant endocrine treatment with tamoxifen can be used in case of high-risk tumors.

## Future perspectives

Future research needs to identify the subgroup of women that will have benefit of extended endocrine treatment. In designing a therapeutic strategy to prevent disease recurrence, it is necessary to not only have knowledge about the total risk of relapse but also to ascertain when recurrence is most likely to occur and when this risk becomes minimal. For this purpose, annual hazard rates could be used. Annual hazard rates describe the changes in the risk of recurrence over time. Instead of simply estimating the overall course of disease, they emphasize when a relapse occurs. When looking at the annual hazard rate curves of women with hormone receptor-positive breast cancer, it comes across that recurrences occur even more than 10 years after the initial diagnosis. Dignam and colleagues presented the annual recurrence hazard for women with node negative early breast cancer that had undergone surgery without subsequent systemic adjuvant treatment [53]. The annual hazard rate for patients with hormone receptor negative tumors reached a peak around 18 months and diminished rapidly afterwards. In the hormone receptor-positive group, this peak appeared slightly later but had a less rapid decrease and did not diminish totally during a follow-up of 12 years. Considering an annual risk of distant recurrence remains 1–2% for at least 15–20 years after diagnosis of hormone receptor-positive breast cancer, even after 5 years of endocrine therapy, extended adjuvant therapy may seem a logical approach [24••]. However, from the trials on extended adjuvant endocrine therapy, it is suggested that these have in general a larger impact on secondary breast cancers and loco-regional recurrences than on distant recurrences. Moreover, it is debated whether the effect size might be larger for extended adjuvant endocrine therapy in those who received initially tamoxifen than in those who received initially AIs. Hence, extending endocrine therapy seems not to be the solution for the observed late distant recurrences.

Moreover, many women are treated with endocrine agents who will never develop metastases. Consequently, they unnecessarily suffer from side effects that influence their quality of life. Therefore, it is important to identify those women with a high risk of relapse and who will have maximum benefit from extended endocrine treatment. For this purpose, several strategies could be used. Firstly, clinical studies investigating endocrine therapy should divide women with hormone receptor-positive breast cancer in luminal A and luminal B subgroups. Luminal B breast cancer has been reported to have lower expression of hormone receptors, higher expression of proliferation markers, and higher histologic grade than luminal A, all exhibiting to a worse prognosis [54]. Furthermore, luminal B breast cancer has a distinct profile of response to endocrine therapy and chemotherapy [54].

The use of several molecular risk scores was approved for use in decision-making concerning adjuvant chemotherapy, however, if these scores can also be used to guide decisions on extended endocrine therapy is not sufficiently clear yet [55]. Nevertheless, the TransATAC trial showed promising results in predicting which women had a low risk of developing distant recurrences 5 to 10 years after breast cancer diagnosis, thereby identifying the women in who extended therapy is not justified [56, 57].

Furthermore, several studies are now combining endocrine therapy with a targeted drug, such as mTOR inhibition or CDK 4/6 inhibition. Much is expected from these combinations, although toxicity is like significantly worse which again can compromise compliance and indirectly efficacy. For that reasons, most studies have chosen to select only high-risk patients based on tumor size, nodal status, and/or histological grade. The on-going trials on the adjuvant endocrine treatment in postmenopausal women with early breast cancer are presented in Table 2.

**Table 2:** On-going clinical trials on adjuvant endocrine treatment in postmenopausal women with early breast cancer

Study acronym Trial ID number Phase country	Sample size (*n*)	Purpose	Inclusion criteria	Endocrine therapy before randomization (years)	Treatment arms	Outcome measures	First results expected
Duration endocrine treatment							
GIM-4-LEAD NCT01064635 Phase III Italy	4050	Comparing the efficacy of different regimens of L in postmenopausal women with stage I, II, or III BC previously treated with T	Postmenopausal women with HR+ BC stage I–III. Any nodal stage. No metastases. Completed initial T treatment.	2–3 years T	1) 2–3 years L 2) 5 years L	OS Safety	2015
ABCSG 16 SALSA NCT00295620 Phase III Austria	3486	Efficacy of a further 2 years vs. a further 5 years of adjuvant treatment with A after initial 5 years of adjuvant endocrine therapy	Postmenopausal women with HR+ BC Any nodal stage No metastases Completed initial anti-hormonal treatment	5 years of any endocrine therapy	1) 2 years A 2) 5 years A	DFS OS Fracture occurrence Secondary carcinoma Contralateral BC	2019
SOLE/ABCSG 35–07 NCT00553410 Phase III International	4800	Continuous L versus intermittent L in postmenopausal women with BC who received 4–6 years of endocrine therapy	Postmenopausal women with HR+ BC Any nodal stage No metastases Completed initial endocrine treatment < 12 months ago	4–6 years of any endocrine therapy	1) 5 years L continuously 2) 5 years intermittent L (4 × 9 months, 1 × 12 months)	DFS OS Distant DFS BC free interval Second malignancies Deaths without prior cancer events Adverse events	2021
MINDACT NCT00433589 Phase III International	6589	Comparing the efficacy of 7 years of L with 2 years of T followed by 5 years of L	Postmenopausal women with HR+ BC 0–3 positive lymph nodes No metastases	none	1) 7 years L 2) 2 years T – 5 years L	DFS OS Safety	Unknown
N-SAS-BC-05 JPRN-UMIN000000818	2500	Comparing the efficacy of 5 year A after either 5 years of A or 5 years of sequential therapy with T followed by A	Postmenopausal women with HR+ BC Any nodal stage No metastases	5 years of A or 5 years of sequential therapy (T followed by A)	1) 5 years A 2) no additional endocrine treatment	DFS OS DDFS Adverse events QALY HRQOL	Unknown
Sequenced treatment versus monotherapy							
GIM-3-FATA NCT00541086 Phase III Italy	10,000	Evaluate the efficacy of sequenced treatment versus AI monotherapy	Postmenopausal women with HR+ BC, stage I–III Any nodal stage No metastases Completed initial endocrine treatment < 2 years ago	none	1) 5 years A, E, or L monotherapy 2) 2.5 years T – 2.5 years A, E, or L	DFS OS DDFS Contralateral BC BC free interval Second malignancy Effects on lipid profile Toxicity	2018
Sequential or concurrent with chemotherapy							
GIM-10-CONSENT NCT02918084 Phase III	1000	Concurrent versus sequential AI for a total of 5 years in postmenopausal patients receiving adjuvant chemotherapy for BC	Postmenopausal women with HR+ BC Any lymph node status No metastases	none	1) Adjuvant chemotherapy followed by 5 years AI (sequential) 2) Adjuvant chemotherapy and 5 years AI concurrent	DFS OS Genomic analysis	2028
Comparison of aromatase inhibitors							
FACE NCT00248170 Phase III International	4160	5 years of adjuvant L versus A in postmenopausal women with HR-positive, node positive BC	Postmenopausal women with HR+ BC Positive lymph nodes No metastases Recently underwent surgery	none	1) 5 years A 2) 5 years L	DFS Safety OS DDFS Cancer-specific survival Effect on lipid profile Bone fractures	2018
PHACS NCT01127295 Phase IV France	2000	The correlation between pharmacokinetic and pharmacogenetic parameters of adjuvant endocrine BC treatment, during the first 3 years	Postmenopausal women with HR+ BC Any lymph node status No metastases	none	1) 5 years T 2) 5 years L 3) 5 years A 4) 5 years E	Correlation pharmacokinetic and pharmacogenetic parameters. Relation plasmatic concentrations, effectivity and adverse events. Relation Polymorphisms and relapses.	2019
Predictive factors							
PreFace NCT01908556 Phase IV Germany	3545	Identification of biomarkers that could predict the efficacy of adjuvant L treatment	Postmenopausal women with HR+ BC Any lymph node status No metastases	None	1) 5 years L	DFS OS Prediction of the above by different pharmacogenetic markers on efficacy and side effects	2015
Long-term follow-up							
LATTE NCT01745289 Phase III USA	6000	Evaluate the long-term effects of A (5 years) versus T (5 years) (follow-up ATAC trial)	All women that were included In the ATAC trial which investigated the efficacy of 5 years of A versus T.	5 years A or 5 years T	Follow-up	Long-term RFS	2018
Targeted therapy							
S1207 NCT01674140 Phase III USA	1900	Evaluate adjuvant endocrine therapy with or without 1 year of everolimus in patients with high risk, hormone receptor-positive, and HER2/Neu negative BC	Pre- and postmenopausal women with HR+ BC Her2– N+	None	Any endocrine therapy combined with 1) 1 year everolimus 2) 1 year placebo	IDFS DFS OS Toxicity	2022
UNIRAD NCT01805271 Phase III France	1984	Evaluate the safety and benefit of adding everolimus to adjuvant endocrine therapy of early BC	Pre- and postmenopausal women with HR+ BC Her2– N+	1 year of any endocrine therapy	1) 1 year everolimus 2) 1 year placebo	DFS OS EFS DMFS Secondary cancer	2021
EarLEE-1 NCT03078751 Phase III USA	2000	Evaluate efficacy and safety of ribociclib with endocrine therapy as adjuvant treatment of high-risk early BC	Pre- and postmenopausal women with HR+ BC Her2– AJCC prognostic stage group III	None	Any endocrine therapy combined with 1) 2 years ribociclib 2) 2 years placebo	IDFS RFS OS Qol	2023
EarLEE-2 NCT03081234 Phase III USA	4000	Evaluate efficacy and safety of ribociclib with endocrine therapy as adjuvant treatment of intermediate risk early BC	Pre- and postmenopausal women with HR+ BC Her2– AJCC prognostic stage group II	None	Any endocrine therapy combined with 1) 2 years ribociclib 2) 2 years placebo	IDFS RFS OS Qol	2025
MonarchE	3580	Evaluate efficacy of abemaciclib combined with standard adjuvant endocrine therapy versus standard adjuvant endocrine therapy alone	Pre- and postmenopausal women with HR+ BC Her2 – N+ status and 1 of the following indicating a higher risk of relapse: - 4 or more N+ - Tumor size ≥ 5 cm - Grade 3 histology - Ki67 index of ≥ 20%	None	Standard 5-year adjuvant endocrine treatment with 1) 2 years palbociclib 2) none	IDFS DRFS OS Toxicity	2022
PALLAS NCT02513394 Phase III USA	4600	Evaluate efficacy of palbociclib with standard adjuvant endocrine therapy versus standard adjuvant endocrine therapy alone	Pre- and postmenopausal women with HR+ BC stage II or III Her2-	None	Standard 5 year adjuvant endocrine treatment with 1) 2 years palbociclib 2) none	IDFS DRFS OS LRRFS	2020

*A* anastrozole, *AI* aromatase Inhibitor, *BC* breast cancer, *BCFI* breast cancer-free interval, *DFS* disease-free survival, *DDFS* distant disease-free survival, *DMFS* distant metastases-free survival, *E* exemestane, *EFS* event free survival, *HR* hormone receptor, *IDFS* invasive disease-free survival, *L* letrozole, *LRRFS* local recurrences-free survival, *P* placebo, *OS* overall survival, *QOL* quality of life, *T* tamoxifen

## Conclusions

**Fig. 1:**
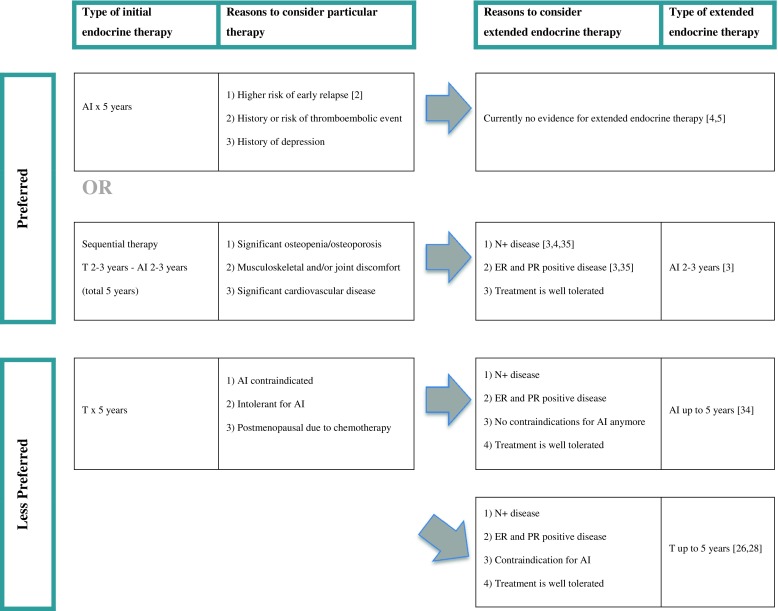
Adjuvant endocrine treatment in postmenopausal women with early stage hormone receptor-positive breast cancer. AI, aromatase inhibitor; T, tamoxifen.

Based on the reviewed literature, we believe both the type and duration of adjuvant endocrine treatment should be personalized based on expected efficacy and tolerability. The identification of subgroups of patients who might benefit from extended endocrine treatment is of great significance. Possibly molecular risk scores will offer more insight hereon in the future. Moreover, it is important to consider quality of life during treatment and other long-term toxicities, such as osteoporosis and cardiovascular diseases that might interfere with overall survival outcome. If a patient tolerates the endocrine treatment well, extended use of hormonal therapy, especially if not initially treated with AIs, could be considered in case of a high-risk tumor that is both ER and PR positive (Fig. 1). But, more targeted treatment approaches are eagerly awaited for from on-going trials.
